# Verbal Perceptual Prompts Facilitate Children’s Sensitivity to False Beliefs

**DOI:** 10.3390/jintelligence12080073

**Published:** 2024-07-27

**Authors:** Qiyu Huang, Xiuli Liu

**Affiliations:** School of Psychology, Northeast Normal University, Changchun 130024, China; huangqy468@nenu.edu.cn

**Keywords:** Theory of Mind, sensitivity to false beliefs, false belief understanding, anticipatory looking paradigm

## Abstract

False belief understanding is always regarded as a milestone of Theory of Mind (ToM), which is an important aspect of social intelligence. Recently, some researchers have suggested the existence of two ToM systems in individuals: one that explicitly guides false belief understanding and another that implicitly directs sensitivity to false beliefs. However, studies on sensitivity to false beliefs have encountered challenges with replicability, and the factors influencing the manifestation of sensitivity to false beliefs remain to be explored. Based on the anticipatory looking task, we investigated whether verbal perceptual prompts could improve children’s performance of sensitivity to false beliefs. Fifty-eight children aged 5 to 6 were randomly assigned tasks with or without verbal perceptual prompts, involving verbal descriptions and explanations of the protagonist’s perceptual state. The findings showed that verbal perceptual prompts could slightly reduce children’s propensity to look at the actual location of the object in false belief situations and increase the likelihood of exhibiting accurate anticipatory looking patterns across false belief and true belief situations. The results suggest that children’s sensitivity to false beliefs may be situation-dependent, yet further investigation is needed to determine which situational factors can most effectively trigger robust sensitivity to false beliefs in children. The results enlighten educational practice, indicating that introducing cues in social environments that convey insights into others’ mental states, akin to the use of learning scaffolding, is advantageous for the development of children’s social cognitive abilities.

## 1. Introduction

### 1.1. The Development of Theory of Mind: Two Systems

In social life, individuals need to understand the thoughts, beliefs, desires, emotions, intentions, and other mental states of others in order to ensure effective social interaction. This ability is called Theory of Mind (ToM), also referred to as mind reading (Slaughter and Perez-Zapata 2014) or mentalizing (Barone and Gomila 2021; Frith and Frith 2006). Using ToM, individuals can interpret and predict the behaviors of others (Apperly and Butterfill 2009; Premack and Woodruff 1978).

Beliefs are an important mental state, referring to the attitude of believing that an individual holds towards the content of a certain proposition (Apperly and Butterfill 2009; Barone and Gomila 2021). For example, “The toy is in Box 1” represents the content of a proposition, and “A believes the toy is in Box 1” represents a belief of A. Depending on whether the propositional content corresponds to objective reality, beliefs can be correct or incorrect. Therefore, a belief is merely a mental representation of an objective reality; it may not accurately reflect objective reality but is perceived by its holder to be in accordance with the reality (Tomasello 2018). A belief that corresponds with objective reality is called a true belief (TB), and a belief that does not correspond with the objective reality is called a false belief (FB).

In the development of ToM, the understanding of others’ false beliefs is considered a milestone (He et al. 2012; Kammermeier and Paulus 2018; Kristen-Antonow et al. 2019; Poulin-Dubois et al. 2023; Yott and Poulin-Dubois 2012). This is because a thorough comprehension of false beliefs means grasping the representational nature of mental states, recognizing that individuals can correctly represent reality as well as misrepresent it and that their behaviors are guided by internal mental states rather than external objective reality. Therefore, individuals may engage in behaviors that correspond to reality or make mistakes that do not align with reality (Wellman et al. 2001).

Research on children’s understanding of false beliefs nearly achieved a consensus in the past that children begin to predict that others holding false beliefs will engage in erroneous actions only in the middle to late stages of preschool (Wellman et al. 2001). This consensus is based on the results of direct false belief tasks, also known as standard, elicited response, or explicit false belief tasks (Barone and Gomila 2021; Low and Perner 2012). These tasks typically involve telling a story to participants about a protagonist who forms a false belief, end with a direct question about the content of the protagonist’s false belief or the prediction of the protagonist’s behavior, requiring participants to respond verbally (He et al. 2012). For instance, a story about location transfer is told to children—Protagonist A placed a toy in Box 1 and then left; in the absence of Protagonist A, Protagonist B moved the toy from Box 1 to Box 2; thereafter, Protagonist A returned. When asked, “Where will Protagonist A look for the toy?”, only older children around the age of 4 or 5 are likely to answer correctly: “Protagonist A will look for the toy in Box 1 because Protagonist A believes the toy is still in Box 1”. Younger children, in contrast, tend to respond based on the actual location of the toy.

However, research that employs indirect false belief tasks has drawn researchers’ attention to sensitivity to false beliefs. Indirect false belief tasks, also known as spontaneous response or implicit false belief tasks (Baillargeon et al. 2010; Low and Perner 2012; Scott and Baillargeon 2009), do not require participants to directly verbally answer questions about the content of the protagonist’s false belief or the behavior of the protagonist who holds a false belief. Instead, they focus on participants’ spontaneous behavioral responses in belief scenarios, such as anticipatory looking, which refers to the behavior of looking ahead to a specific location before an agent acts. For example, when the protagonist is about to search for the toy in Box 1, participants shift their gaze to Box 1 in advance; this is commonly considered a correct anticipatory looking response (Barrett et al. 2013; He et al. 2012; Scott and Baillargeon 2009). Many studies using these indirect false belief tasks have shown that children, even if they are unable to verbally predict the behavior of others holding false beliefs, can spontaneously exhibit adaptive looking or helping behaviors that are congruent with others’ belief states, thus demonstrating sensitivity to false beliefs. For example, they looked ahead to the location that the mistaken protagonist intended to act on (Clements and Perner 1994; He et al. 2012; Meristo et al. 2012; Senju et al. 2011; Southgate et al. 2007; Surian and Geraci 2012; Thoermer et al. 2012), preferred to look at the outcome that matched the protagonist’s false belief (Barrett et al. 2013; Scott et al. 2012), could detect the conflict between the presented outcome and the protagonist’s false belief (Onishi and Baillargeon 2005; Scott et al. 2012; Song and Baillargeon 2008; Song et al. 2008; Surian et al. 2007; Träuble et al. 2010; Yott and Poulin-Dubois 2012), exhibited different helping behaviors towards others with different belief states (Buttelmann et al. 2009, 2014, 2015; Southgate et al. 2010), informed others with false beliefs about the unexpected events in advance (Knudsen and Liszkowski 2012a, 2012b), and so on. Furthermore, some studies have shown that non-human animals can also exhibit sensitivity to false beliefs (Buttelmann et al. 2017; Hayashi et al. 2020; Krupenye et al. 2016; Lonardo et al. 2021).

Regarding the phenomenon of sensitivity to false beliefs mentioned above, some researchers have proposed the Two-Systems Account of the development of ToM (Apperly and Butterfill 2009; Butterfill and Apperly 2013). According to this theory, ToM consists of two systems that develop independently: one is a full-blown Theory of Mind, acquired in the later stages of early childhood, which allows for explicit and flexible expression, as demonstrated by the results of standard false belief tasks; the other is a minimal Theory of Mind, acquired early in life and persisting throughout one’s life, which allows for implicit and efficient expression, as demonstrated by the results of indirect false belief tasks. Thus, unlike one-system views, which propose that early developmental abilities are replaced by later ones (Baillargeon et al. 2010; Ruffman 2014), the Two-Systems Account posits that early sensitivity to false beliefs is not replaced by a more mature understanding of false beliefs that develops later in life. Studies on individuals with autism found that they performed at the level of typically developing individuals on explicit Theory of Mind tasks but exhibited persistent impairment in performance on implicit false belief tasks (Schneider et al. 2013; Schuwerk et al. 2015, 2016; Senju et al. 2010). Neuroimaging studies demonstrated that the brain regions activated by implicit false belief tasks and explicit false belief tasks overlapped, particularly within the temporoparietal junction (Bardi et al. 2017; Hyde et al. 2015; Naughtin et al. 2017). However, the activations were not entirely congruent, and there might have been functional differences (Biervoye et al. 2016; Boccadoro et al. 2019; Nijhof et al. 2018). These findings of dissociation in performance support the view that there are two separate ToM systems that develop independently.

### 1.2. The Failed Replication of Sensitivity to False Beliefs

In recent years, the replicability of the phenomenon of sensitivity to false beliefs has been challenged. Many confirmatory studies have failed to fully replicate the positive results previously reported on sensitivity to false beliefs (Dörrenberg et al. 2018; Kulke et al. 2019; Kulke and Rakoczy 2019; Powell et al. 2018; Schuwerk et al. 2018), indicating that replicating the phenomenon of sensitivity to false beliefs is challenging, and the reasons for this remain a subject of debate and exploration. One perspective suggests that the negative replication results may suggest that sensitivity to false beliefs does not truly exist, or, if it does, its strength is weak (Schuwerk et al. 2018). An alternative perspective posits that sensitivity to false beliefs is indeed real and robust and that negative replication results merely indicate issues with the methods used to detect it. For instance, experimental factors such as whether experimental procedures align with the original ones, whether the context is clear enough for participants to form expectations, and whether participants have sufficient time to process relevant information, as well as differences in participants’ attention and motivation during the experiment, can influence the detection of sensitivity to false beliefs (Baillargeon et al. 2018).

In response to the challenges in replicating sensitivity to false beliefs, research should not hastily dismiss its existence. A more prudent and important approach is to analyze the reasons behind the current mixed results and to identify factors that may facilitate the manifestation of individuals’ sensitivity to false beliefs. This serves as the starting point for the present study.

### 1.3. The Verbal Prompts and Sensitivity to False Beliefs

Verbal prompts in belief tasks are linguistic cues to facilitate the comprehension of scenarios or to direct attention to the protagonist’s mental state. If verbal prompts are crafted to target the protagonist’s perceptual state, for instance, indicating whether the protagonist has access to key events, they are referred to as verbal perceptual prompts. However, studies using indirect false belief tasks to investigate the precise role of prompts in individuals’ sensitivity to false beliefs are quite limited.

From a broader perspective on ToM, studies using direct false belief tasks to measure children’s understanding of false beliefs have revealed that their performance on false belief tasks exhibits a certain degree of variability (Wellman et al. 2001). Compared to the classic question of where the protagonist would look for the object without any prompts, children were more inclined to provide the correct answer when the question was accompanied by prompts suggesting where the protagonist would look for the object “first” (Siegal and Beattie 1991). Similarly, when the classic question was altered to imply a difference in perspectives, such as “You and I know that the toy is in Box 1, where does the protagonist think it is?”, 3-year-old children could potentially provide the correct answer (Hansen 2010). In a related finding, children were more likely to comprehend sentences stating others’ false beliefs when the strong negatively biased mental verb “falsely think” (yiwei/以为 in Chinese) was used instead of the neutral mental verb “think” (juede/觉得 in Chinese) (Zhang and Zhou 2021). These findings indicate that specific linguistic prompts can facilitate children’s understanding of others’ false beliefs.

Similar to the effect observed in children’s performance on direct false belief tasks, do relevant verbal prompts also promote children’s sensitivity to false beliefs as manifested in performance on indirect false belief tasks? In a study by Kulke and Rakoczy (2019), the researchers investigated the role of verbal narration in preschoolers’ sensitivity to false beliefs in two frequently repeated anticipatory looking tasks (Southgate et al. 2007; Surian and Geraci 2012). Compared with the original task version without verbal narration, the verbal narration task version included a synchronized narrating voice that described the events as they happened in the scene, such as “This is Teddy. Teddy brought a ball. He puts the ball in the box… Oh, the phone rings!” The results showed that the addition of verbal narration to the stimulus video only slightly improved the children’s tendency to look at the correct location in one of the false belief conditions of the Southgate task (Southgate et al. 2007), as indicated by fixation duration metrics. Children still did not demonstrate a significant preference for longer looking at the correct location in that task. Furthermore, in the Surian and Geraci task, when compared to the results without verbal narration, the proportion of children looking at the box containing the object (an incorrect response commonly considered) rather than the empty box (a correct response commonly considered) increased under the false belief scenarios with verbal narration. Based on these findings, the researchers concluded that the effect of verbal narration was very limited, and the phenomenon of sensitivity to false beliefs was not replicated in this study. Nevertheless, there is room for further exploration.

Firstly, in the task by Kulke and Rakoczy (2019), the additional verbal narration mentioned the protagonist’s goal state of what the protagonist intended to do. However, during the critical location transfer event, there was no narration regarding the protagonist’s perceptual state of whether the protagonist had access to the subsequent event, which was a crucial determinant in the formation of the protagonist’s belief state. Thus, the observed weak effect of verbal narration might merely indicate that children indeed had no difficulty in understanding the plot of the contexts and the protagonist’s intended actions. This leads to the following question: can providing precise verbal prompts that describe the protagonist’s perceptual state effectively facilitate children’s sensitivity to false beliefs? This is the main question that the present study aims to investigate.

Secondly, verbal narration was found to increase the mean proportion of time that children spent looking at the position with the object, rather than at the empty position, in the Surian and Geraci task (Kulke and Rakoczy 2019). This finding appears to suggest that verbal narration may actually hinder the manifestation of children’s sensitivity to false beliefs. However, if we move beyond the common standard in anticipatory looking tasks, which favors looking at the empty location in false belief scenarios, and instead consider the exhibition of different looking responses across false belief and true belief scenarios, involving distinguishing between false beliefs from true beliefs, as an indication of sensitivity to false beliefs, then the direction of the effect of verbal narration described above can also be interpreted as facilitative. The results showed that regardless of the presence or absence of verbal narration, children’s tendency to look at the location with the object in the false belief scenarios of the Surian and Geraci task was significant. In contrast, children did not show such a significant tendency in the true belief scenarios, where the protagonist witnessed the event of location transfer, thus forming a belief consistent with objective reality. This demonstrated differences in children’s looking responses across different belief state scenarios to some extent. Children behaved differently depending on the beliefs held by the protagonist, demonstrating a form of sensitivity to false beliefs. Although children in both false belief and true belief scenarios tended to look longer at the location with the object, the presence of verbal narration significantly increased this tendency in false belief scenarios without altering it in true belief scenarios. As a result, there was a trend for the difference in looking tendencies between false belief and true belief scenarios to increase when verbal narration was present. From a differential perspective, the role of verbal narration could be seen as amplifying the differences in children’s looking responses in different belief state scenarios, which could be interpreted as facilitating sensitivity to false beliefs. We hypothesize that sensitivity to false beliefs may manifest in various ways, and thus, the detection of sensitivity to false beliefs requires the use of more sensitive indicators. The difficulty in replicating previously reported results of sensitivity to false beliefs may be related to the choice of indicators used in those studies. This issue is precisely what the present study attempts to explore regarding task analysis methodology.

### 1.4. The Present Study

In summary, the present study investigates whether verbal prompts emphasizing the protagonist’s perceptual state can facilitate children’s sensitivity to false beliefs and explores the various ways children’s sensitivity to false beliefs is likely to manifest.

Consistent with Kulke and Rakoczy (2019), the present study also utilized the anticipatory looking task, which centered on children’s gaze behavior in belief scenarios. However, as previously mentioned, experimental factors such as the clarity of the context, the allocation of sufficient time for information processing, and variations in attention and motivation may influence the detection of sensitivity to false beliefs (Baillargeon et al. 2018). Therefore, the present study attempts to refine the anticipatory looking task.

A revised version of the anticipatory looking task was developed. It employed animated stimuli, which are more engaging for children, and established clear target indicators to improve the clarity of the protagonist’s goal. The task also included a pause after each event, allowing children time to respond. Thus, the task aimed to maintain children’s attention and motivation to participate. Regarding verbal prompts, instead of offering a narrative that described the surface plot, the task with prompts used concise verbal perceptual prompts that specified whether the protagonist had access to the upcoming events. Delivering such messages at the critical initial stage of belief formation, the task with prompts drew children’s attention to the protagonist’s perceptual state. Consequently, it might enhance children’s attention to the protagonist’s belief state and facilitate their sensitivity to false beliefs.

Contrary to the single-system perspective theories that suggest sensitivity to false beliefs is replaced by the understanding of false beliefs in later preschool stages (Baillargeon et al. 2010; Ruffman 2014), the Two-Systems Account of ToM posits that once sensitivity to false beliefs emerges, it persists throughout life (Apperly and Butterfill 2009; Butterfill and Apperly 2013). This implies that sensitivity to false beliefs continues to exist after the development of a mature understanding of false beliefs. It is reasonable to investigate children’s sensitivity to false beliefs in the later stages of preschool. Furthermore, with more advanced language skills, older children may better understand the meaning of the additional verbal perceptual prompts. Thus, the present study recruited children aged 5 to 6 years, who had a well-developed understanding of false beliefs and strong language skills, as participants.

Besides the common analysis of the proportions of first looks or looking time within a single false belief scenario, the study also examined the response patterns of the same participant across false belief and true belief scenarios. The focus is on discerning whether children can distinguish between scenarios involving false beliefs and those involving true beliefs. This approach aims to explore whether children’s sensitivity to false beliefs is more readily reflected in the differential responses between different belief scenarios, beyond the common indicators within a single false belief scenario.

The hypothesis of the present study is that verbal perceptual prompts can facilitate children’s sensitivity to false beliefs, and this facilitative effect may be more evident in children’s differential responses between false belief and true belief scenarios.

## 2. Materials and Methods

### 2.1. Participants

Fifty-eight children aged 5 to 6 years (*M* = 6.3 years, range = 5.6 to 6.8 years, *SD* = 0.30 years, 28 girls and 30 boys) successfully completed the experimental tasks. Three additional children also provided data but were excluded from the analyses due to failure to show anticipatory looking in at least one test trial. Children were randomly assigned to either the no-verbal-perceptual-prompts group (*N* = 30, *M* = 6.3 years, range = 5.8 to 6.7 years, *SD* = 0.29 years, 13 girls and 17 boys) or the verbal-perceptual-prompts group (*N* = 28, *M* = 6.4 years, range = 5.6 to 6.8 years, *SD* = 0.31 years, 15 girls and 13 boys). All participants were recruited from Qingshen County Kindergarten in Meishan City, Sichuan Province, China. They spoke Mandarin as their first language and were all of Han ethnicity. None of them had been diagnosed with any language developmental delays or psychological disorders. The experimental protocol was approved, and informed consent was obtained from the teachers and parents of the participating children.

### 2.2. Apparatus

A Tobii X3-120 desktop eye tracker (sampling rate 120 Hz; Tobii, Stockholm, Sweden) was used to record the participants’ gaze behavior, while a portable Logitech webcam (C920 Pro; Logitech, Lausanne, Switzerland) in front of the participants synchronized the recording of the experimental process. Stimulus presentation and data collection and storage were conducted on a 15.6-inch laptop using Tobii Studio Pro software version 4.3.8.

### 2.3. Stimuli

The stimuli were two-dimensional animated videos created using Flash, with a resolution of 1920 × 1024 pixels. The animations were adapted from common anticipatory looking task scenarios. The stimuli comprised familiarization trials followed by test trials. The basic scene for each trial was identical. In each trial, participants watched a simplified cartoon anthropomorphic protagonist searching for a geometric object indicated by a sign placed at the top of the central wall. To attract and maintain participants’ attention, each trial featured a different protagonist with distinct colors and a different target object varying in color and shape. Additionally, cartoon sound effects mimicking blinking and walking accompanied the protagonist’s behaviors.

**Figure 1:**
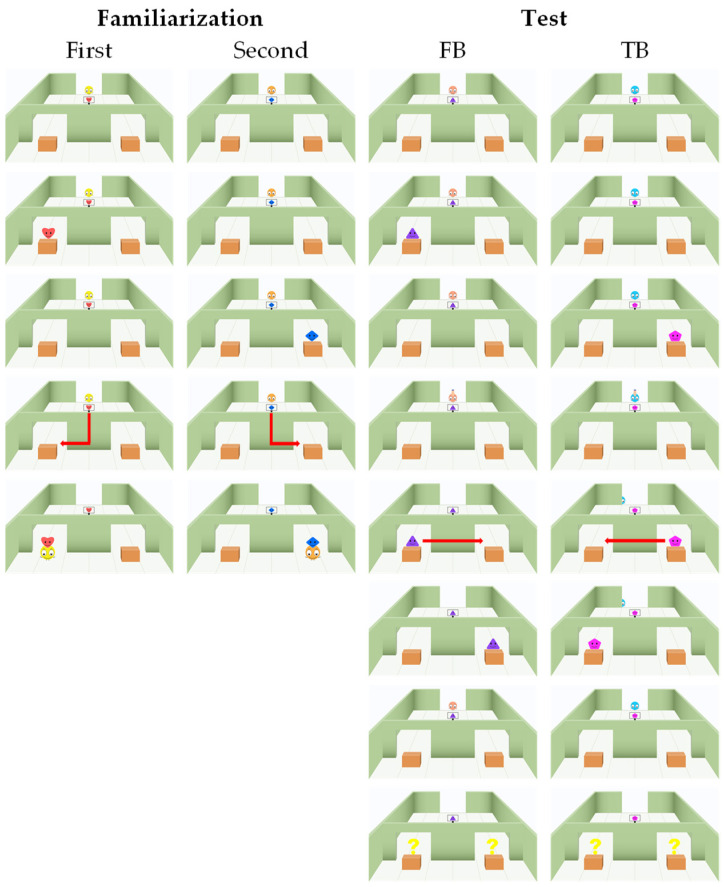
Selected still frames depicting key events from two familiarization trials and two test trials. To sustain the interest of participants, different protagonists and target objects were utilized in each trial. The arrows in the figure indicate the movement trajectories of the protagonists or the target objects. In the false belief (FB) condition, the protagonist was completely moved out of sight behind the wall by a hand, resulting in no access to the subsequent location transfer event. In the true belief (TB) condition, the protagonist was partially concealed by the wall, with only one eye obscured, thereby witnessing the subsequent event.

As illustrated in Figure 1, in the familiarization trials, the protagonist noticed a sign depicting the target object and displayed a smiling expression. Subsequently, the protagonist looked left and right in sequence. The target object would “emerge” from one of the boxes before the protagonist’s looking, appearing above the box. After the protagonist’s attention returned to the center, a “ding” sound (0.5 s), serving as an action prompt, rang out in the scene. Following a 0.5 s interval, the protagonist began to move forward in a straight line. After 1.5 s, the protagonist had completely moved behind the central wall and disappeared from the participant’s view, but the walking sound accompanying the protagonist’s movement continued to play, suggesting that the protagonist was still in motion. After a 2.5 s interval, the protagonist moved horizontally and appeared at the doorway on the side where the target object was located. The box then opened automatically and disappeared, revealing the target object. With a smiling expression, the protagonist moved forward and “balanced” the target object on its head, indicating that the protagonist had successfully found the target object.

In the test trials, the initial scenario was identical to that of the familiarization trials, in which the protagonist observed which side box contained the target object and then gazed straight ahead. The difference was that before the action prompt sound was played, a critical perceptual event occurred—a hand extended downward from above the background wall’s doorway and “pulled” the protagonist into movement. Under the false belief condition (FB condition), the protagonist was completely moved behind the background wall, which fully obstructed the protagonist and the line of sight. Under the true belief condition (TB condition), the protagonist was also moved, but only half of the body was blocked by the background ball, allowing one eye to remain visible, enabling the protagonist to observe the subsequent events. Subsequently, under both belief conditions, the same critical location transfer event occurred—the target object emerged from one side box and moved to the other. After the critical location transfer event was fully concluded, the protagonist returned to the original location. After a 2 s interval, similar to the familiarization trial, the action prompt sound was played, and the protagonist began to act. The difference then was that after the protagonist was completely obscured by the central wall for 2.5 s, the animation did not reveal which door the protagonist emerged from. Instead, question marks appeared simultaneously at both doorways, avoiding the presentation of the outcome affecting the participant’s subsequent performance.

### 2.4. Design and Procedure

The study employed a mixed experimental design, with participants randomly assigned to either the group with verbal perceptual prompts or the group without such prompts. Each participant underwent one false belief test and one true belief test. The design of the verbal perceptual prompts varied across different groups and test trials. For the group with verbal perceptual prompts, after each critical perceptual event in the video, the experimenter, depending on the specific test trial, narrated sentences (a) or (b) as if providing a voice-over. In contrast, for the group without verbal perceptual prompts, the experimenter did not provide any additional prompts, allowing the participants to watch the video independently.

(a) Prompts in the FB trial (only for the prompt group): the protagonist has been completely moved away and cannot see what happens next (Xiaoren bei wanquan yizou le, ta kanbudao jiexialai fasheng le shenme/小人被完全移走了，它看不到接下来发生了什么).

(b) Prompts in the TB trial (only for the prompt group): the protagonist has not been completely moved away and is peeking (Xiaoren meiyou bei wanquan yizou, ta zai toukan/小人没有被完全移走，它在偷看).

Before the official start of the experiment, participants were seated in front of a computer screen. The experimenter adjusted the distance between the participant and the screen and performed a medium-speed 5-point calibration on the participant using the Tobii Studio software. Once the calibration met the standards, the experiment began. The screen initially displayed an image of the protagonist standing at the doorway of the background wall. The experimenter instructed the participant: “You will watch some animations about the protagonist. Please observe carefully what happens and you can also try to guess which door the protagonist will come out of next.” Then, each participant underwent two familiarization trials and two test trials in sequence. The order of the false belief and true belief trials was counterbalanced across participants (sequence FB-TB or FB-TB). To maintain the participants’ attention, the initial positions of the object in the two test trials were different, thus ensuring the same final correct response position for both trials. To avoid having the same correct response position for three consecutive trials, the correct response position for the second familiarization trial was different from the subsequent test trial. Consequently, the sequence of correct response positions across the two familiarization trials and the two test trials was either left–right–left–left or right–left–right–right. This sequence of correct response positions was also counterbalanced across participants. At the end of the test trials, after the question marks appeared at the central wall doorway, the experimenter asked the participant: “Which door will the protagonist come out of next?” The trial ended when the participant had verbally answered the question or after a 30 s waiting period. After the experiment, participants received a small sticker as a token of appreciation for their participation.

### 2.5. Coding

**Figure 2:**
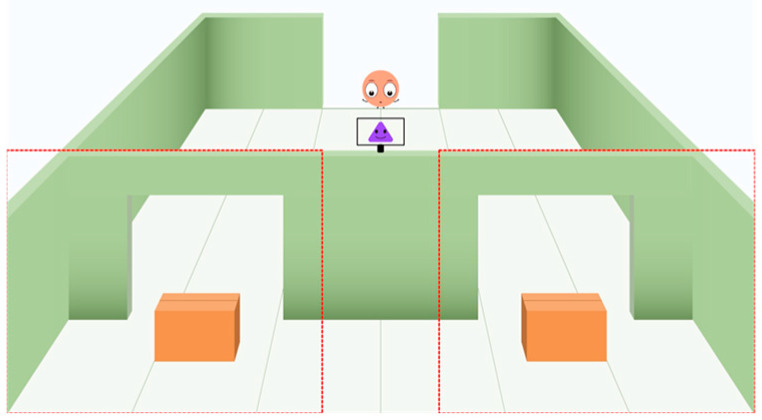
The areas of interest (AOIs), indicated by dashed lines, used to encode participants’ anticipatory looking responses.

For the eye-tracking metrics, the eye movement data were directly exported from Tobii Studio Pro. The analysis focused on a 7 s period of interest (POI) during the test trial, starting from the frame where the protagonist returned to the initial position and ending at the frame where the question marks appeared in the scene. The looking durations and the first look to two areas of interest (AOIs)—the empty box position without the object (the empty position/the target position) and the full box position with the object (the full position/the non-target position), as shown in Figure 2—were analyzed. Based on the looking durations, the difference looking score (DLS) for each participant was calculated by subtracting the total looking time at the full position from the total looking time at the empty position and then dividing this value by the sum of the total looking times at both positions. Based on the first looks, the proportion of participants who first looked at the empty position was calculated. Additionally, each participant’s DLS-based gaze pattern and first look-based gaze pattern across the FB and TB conditions were coded. A positive DLS, indicating longer looking at the empty position, or a first look toward the empty position under the FB condition was coded as 1; otherwise, it was coded as 0. A negative DLS, indicating longer looking at the full position, or a first look toward the full position under the TB condition was coded as 1; otherwise, it was coded as 0. Using the coding sequence with FB followed by TB, four response patterns were identified: the 1-1 and 0-0 patterns indicated differential patterns, showing that the participant’s gaze behaviors differed between the FB and TB conditions; and the 0-1 and 1-0 patterns indicated non-differential patterns, showing that the participant’s gaze behaviors were consistent across the FB and TB conditions. Participants who observed the critical perceptual event, the critical location transfer event, and exhibited anticipatory looking during the POI were included in subsequent analysis of eye movement behavior.

For the verbal response metrics, participants’ verbal answers concerning the position from which the protagonist would emerge were coded based on the experimental video recordings. The video playback began only from the final verbal response phase, and the coders were unaware of the specific experimental design details. Based on the verbal answers, the proportion of participants who verbally answered that the protagonist would come out from the empty position was calculated. Additionally, following the same coding standards as for the gaze patterns, participants’ verbal response patterns across the FB and TB conditions were also coded. Only if participants provided a clear verbal answer were their responses included in the verbal behavior analysis.

## 3. Results

### 3.1. DLS

**Figure 3:**
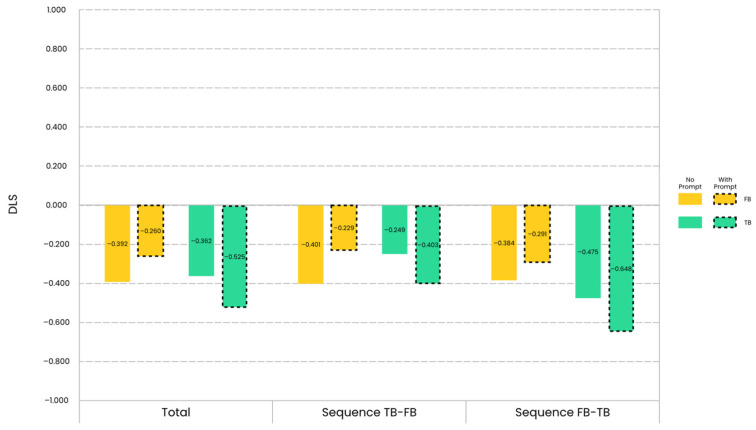
Mean differential looking scores for the no-prompt group and the prompt group across trial sequences and belief conditions. Under the FB condition, the tendency to look longer at the full position was weaker for the prompt group (indicated by dashed lines) than for the no-prompt group. Conversely, under the TB condition, the tendency to look longer at the full position was stronger for the prompt group than for the no-prompt group.

Descriptive results of the children’s DLS are illustrated in Figure 3.

The direct comparison of DLS between the prompt group and the no-prompt group did not reveal any significant group effect. Whether under the FB condition or under the TB condition, there was no significant difference in DLS between the two groups in any sequence, and the Bayesian test demonstrated anecdotal to moderate evidence for the null hypothesis (Mann–Whitney U Test, *p_s_* > 0.05; 2.884 ≤ BF_01_ ≤ 3.806). The direct comparison of DLS between the FB condition and the TB condition also did not reveal any significant belief effect. Regardless of whether for the prompt group or for the no-prompt group, there was no significant difference in DLS between the two belief conditions in any sequence, and the Bayesian test indicated anecdotal to moderate evidence for the null hypothesis (Wilcoxon Signed Ranks Test, *p_s_* > 0.05; 2.098 ≤ BF_01_ ≤ 6.961). However, the separate group comparison of DLS to zero indicated a group effect. The details are as follows.

In terms of the FB condition, no matter in the sequence TB-FB or in the sequence FB-TB, the DLS for the prompt group was not significantly different from zero, and there was anecdotal evidence for the null hypothesis (One-Sample Wilcoxon Signed Rank Test, *p_sequence TB-FB_* = 0.157, *p_sequence FB-TB_* = 0.220; BF_01 *sequence TB-FB*_ = 2.129, BF_01 *sequence FB-TB*_ = 1.887). However, the DLS for the no-prompt group was significantly less than zero, and there was anecdotal (in the sequence TB-FB) or moderate (in the sequence FB-TB) evidence for the alternative hypothesis (One-Sample Wilcoxon Signed Rank Test, *p_sequence TB-FB_* = 0.039, *p_sequence FB-TB_* = 0.011; BF_01 *sequence TB-FB*_ = 0.599, BF_01 *sequence FB-TB*_ = 0.264). These results, combined with the descriptive statistics, indicated that although children generally tended to look longer at the full position under the FB condition, this tendency was weaker in children from the prompt group. The verbal perceptual prompts had reduced the extent to which children preferentially looked at the commonly considered incorrect location under the FB condition.

Similarly, in terms of the TB condition, no matter in the sequence TB-FB or in the sequence FB-TB, the DLS for the prompt group was significantly less than zero, and there was anecdotal (in the sequence TB-FB) or very strong (in the sequence FB-TB) evidence for the alternative hypothesis (One-Sample Wilcoxon Signed Rank Test, *p_sequence TB-FB_* = 0.030, *p_sequence FB-TB_* = 0.002; BF_01 *sequence TB-FB*_ = 0.378, BF_01 *sequence FB-TB*_ = 0.022). However, the DLS for the no-prompt group was significantly less than zero only in the sequence FB-TB, and there was anecdotal evidence for the null hypothesis in the sequence TB-FB and moderate evidence for the alternative hypothesis in the sequence FB-TB (One-Sample Wilcoxon Signed Rank Test, *p_sequence TB-FB_* = 0.153, *p_sequence FB-TB_* = 0.014; BF_01 *sequence TB-FB*_ = 2.063, BF_01 *sequence FB-TB*_ = 0.179). These results, in conjunction with the descriptive statistics, suggested that although children tended to look longer at the full position under the TB condition, this tendency was stronger in children from the prompt group. The verbal perceptual prompts had increased the extent to which children preferentially looked at the commonly considered correct location under the TB condition.

Thus, by combining the above results from the FB and TB conditions, it was concluded that the verbal perceptual prompts simultaneously increased the likelihood of children making the anticipatory looking responses that were commonly considered correct under both belief conditions.

When organized at the level of each group, the above results also showed a belief effect. The details are as follows.

For the prompt group, the belief effect lay in that, no matter in the sequence TB-FB or in the sequence FB-TB, the DLS under the FB condition was not significantly different from zero, and there was anecdotal evidence for the null hypothesis (One-Sample Wilcoxon Signed Rank Test, *p_sequence TB-FB_* = 0.157, *p_sequence FB-TB_* = 0.220; BF_01 *sequence TB-FB*_ = 2.129, BF_01 *sequence FB-TB*_ = 1.887). However, the DLS under the TB condition was significantly less than zero, and there was anecdotal (in the sequence TB-FB) or strong (in the sequence FB-TB) evidence for the alternative hypothesis (One-Sample Wilcoxon Signed Rank Test, *p_sequence TB-FB_* = 0.030, *p_sequence FB-TB_* = 0.002; BF_01 *sequence TB-FB*_ = 0.378, BF_01 *sequence FB-TB*_ = 0.022). These results, combined with the descriptive statistics, indicated that for the prompt group, children’s tendency to look longer at the full position was weaker under the FB condition than that under the TB condition. Children from the prompt group differentiated FB from TB, showing a kind of sensitivity to false beliefs both in the sequence TB-FB and in the sequence FB-TB.

For the no-prompt group, the belief effect only lay in the sequence TB-FB. In the sequence TB-FB, the DLS under the FB condition was significantly less than zero, and there was anecdotal evidence for the alternative hypothesis (One-Sample Wilcoxon Signed Rank Test, *p* = 0.039; BF_01_ = 0.599). In contrast, in the sequence TB-FB, the DLS under the TB condition was not significantly different from zero, and there was anecdotal evidence for the null hypothesis (One-Sample Wilcoxon Signed Rank Test, *p* = 0.153, BF_01_ = 2.063). These results, combined with the descriptive statistics, indicated that for the no-prompt group, children’s tendency to look longer at the full position was stronger under the FB condition than that under the TB condition. Children from the no-prompt group differentiated FB from TB in the sequence TB-FB, although the direction of difference was opposite to that of the common anticipatory looking behavior. From a differential perspective, children from the no-prompt group demonstrated a kind of sensitivity to false beliefs, but only in the sequence TB-FB.

### 3.2. Proportion of First Looks at the Empty Position

**Figure 4:**
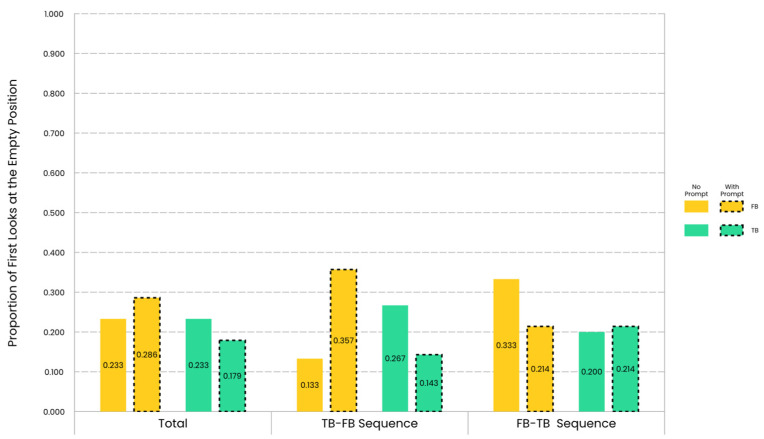
Proportion of first looks at the empty position for the no-prompt group and the prompt group across trial sequences and belief conditions. Under the FB condition, children’s tendency to first look at the empty position was stronger for the prompt group (indicated by dashed lines) than for the no-prompt group in the sequence TB-FB.

Descriptive results of the children’s proportion of first looks at the empty position are illustrated in Figure 4.

As in the analysis of DLS, the direct comparison of the proportion of first looks at the empty position between the prompt group and the no-prompt group did not reveal any significant group effect (Chi-Square Tests, *p_s_* > 0.05). Similarly, the direct comparison of the proportion of first looks at the empty position between the FB condition and the TB condition also did not reveal any significant belief effect (McNemar Test, *p_s_* > 0.05). However, the single group comparison of the proportion of first looks at the empty position to the chance level at 0.5 revealed a group effect and a belief effect. The specific details are as follows.

In terms of the FB condition, in the sequence TB-FB, the proportion of first looks at the empty position for the prompt group was not significantly different from 0.5, and there was anecdotal evidence for the null hypothesis (*χ*^2^ = 1.143, *df* = 1, *p* = 0.285; BF_01_ = 1.385). However, the proportion of first looks at the empty position for the no-prompt group was significantly less than 0.5, and there was strong evidence for the alternative hypothesis (*χ*^2^ = 8.067, *df* = 1, *p* = 0.005; BF_01_ = 0.062). In the sequence FB-TB, the proportion of first looks at the empty position for the prompt group was significantly less than 0.5, and there was moderate evidence for the alternative hypothesis (*χ*^2^ = 4.571, *df* = 1, *p* = 0.033; BF_01_ = 0.315). However, the proportion of first looks at the empty position for the no-prompt group was not significantly different from 0.5, and there was anecdotal evidence for the null hypothesis (*χ*^2^ = 1.667, *df* = 1, *p* = 0.197; BF_01_ = 1.333). These results, combined with the descriptive statistics, indicated that although children generally tended to first look at the full position under the FB condition, this tendency was weaker for the prompt group in the sequence TB-FB and stronger for the prompt group in the sequence FB-TB. In other words, the verbal perceptual prompt influenced children’s first looks, with the direction of this influence varying by trial sequences.

In terms of the TB condition, the group effect only lay in the sequence TB-FB. In the sequence TB-FB, the proportion of first looks at the empty position for the prompt group was significantly less than 0.5, and there was strong evidence for the alternative hypothesis (*χ*^2^ = 7.143, *df* = 1, *p* = 0.008; BF_01_ = 0.097). However, in the sequence TB-FB, the proportion of first looks at the empty position for the no-prompt group was not significantly different from 0.5, and there was only anecdotal evidence for the alternative hypothesis (*χ*^2^ = 3.267, *df* = 1, *p* = 0.071; BF_01_ = 0.567). These results, in conjunction with the descriptive statistics, suggested that although children tended to first look at the full position under the TB condition, this tendency was stronger in children from the prompt group. The verbal perceptual prompt enhanced the tendency for children to first look at the commonly considered correct location under the TB condition.

Thus, by combining the results from the FB and TB conditions, it was concluded that the verbal perceptual prompts simultaneously increased the likelihood of children making the anticipatory looking responses that were commonly considered correct under both belief conditions in the sequence TB-FB. However, the effect was opposite in the sequence FB-TB, as the verbal perceptual prompts decreased the likelihood of children to make the commonly considered correct predictions under the FB condition.

A belief effect also emerged when the results were examined within each group, as detailed below.

For the prompt group, the belief effect only lay in the sequence TB-FB. In the sequence TB-FB, the proportion of first looks at the empty position under the FB condition was not significantly different from 0.5, and there was anecdotal evidence for the null hypothesis (*χ*^2^ = 1.143, *df* = 1, *p* = 0.285; BF_01_ = 1.385). However, in the sequence TB-FB, the proportion of first looks at the empty position under the TB condition was significantly less than 0.5, and there was strong evidence for the alternative hypothesis (*χ*^2^ = 7.143, *df* = 1, *p* = 0.008; BF_01_ = 0.097). These results, combined with the descriptive statistics, indicated that in the sequence TB-FB, children from the prompt group differentiated FB from TB, showing sensitivity to false beliefs only in the sequence TB-FB.

For the no-prompt group, the belief effect lay in both sequences. In the sequence TB-FB, the proportion of first looks at the empty position under the FB condition was significantly less than 0.5, and there was strong evidence for the alternative hypothesis (*χ*^2^ = 8.067, *df* = 1, *p* = 0.005; BF_01_ = 0.062). However, the proportion of first looks at the empty position under the TB condition was not significantly different from 0.5, and there was only anecdotal evidence for the alternative hypothesis (*χ*^2^ = 3.267, *df* = 1, *p* = 0.071; BF_01_ = 0.567). In the sequence FB-TB, the proportion of first looks at the empty position under the FB condition was not significantly different from 0.5, and there was anecdotal evidence for the null hypothesis (*χ*^2^ = 1.667, *df* = 1, *p* = 0.197; BF_01_ = 1.333). However, the proportion of first looks at the empty position under the TB condition was significantly less than 0.5, and there was moderate evidence for the alternative hypothesis (*χ*^2^ = 5.400, *df* = 1, *p* = 0.020; BF_01_ = 0.218). These results, combined with the descriptive statistics, indicated that children from the no-prompt group differentiated FB from TB both in the sequence TB-FB and in the sequence FB-TB, although the directions of difference were opposite. From a differential perspective, children from the no-prompt group demonstrated a kind of sensitivity to false beliefs in both sequences.

### 3.3. Proportion of Verbal Answers to the Empty Position

**Figure 5:**
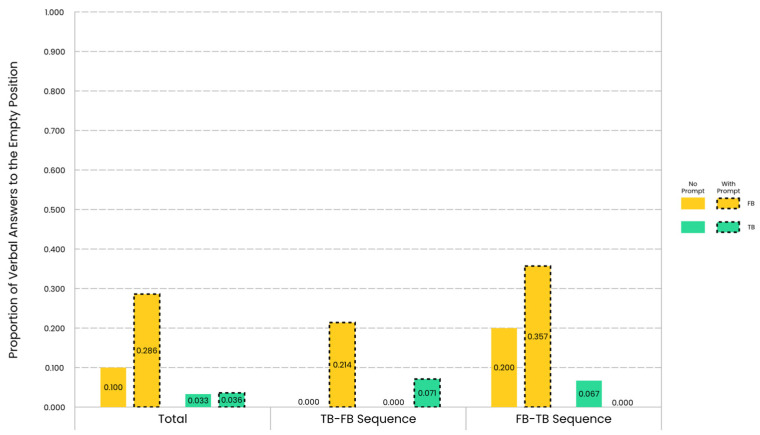
Proportion of verbal answers to the empty position for the no-prompt group and the prompt group across trial sequences and belief conditions. Under the FB condition, children’s tendency to verbally answer that the protagonist would come out from the empty position was stronger for the prompt group (indicated by dashed lines) than for the no-prompt group, especially in the sequence TB-FB.

Descriptive results of the proportion of children’s verbal answers to the empty position are illustrated in Figure 5. This proportion represents the ratio of children who verbally answered that the protagonist would come out from the door behind the empty box.

The direct comparison of the proportion of verbal answers to the empty position between the prompt group and the no-prompt group revealed a marginally significant group effect but only under the FB condition. Under the FB condition, the proportion of verbal answers to the empty position for the prompt group was marginally significantly higher than that for the no-prompt group in the overall analysis (*χ*^2^ = 3.250, *df* = 1, *p* = 0.071; BF_01_ = 0.573) or in the sequence TB-FB (Fisher’s Exact Test, *p* = 0.100; BF_01_ = 0.549), and there was anecdotal evidence for the alternative hypothesis. These results, combined with the descriptive statistics, indicated that the verbal perceptual prompts increased the likelihood for children to make a correct verbal expected response under the FB condition, especially in the sequence TB-FB.

The direct comparison of the proportion of verbal answers to the empty position between the FB condition and the TB condition revealed a belief effect only for the prompt group. For the children in the prompt group, the proportion of verbal answers to the empty position was significantly higher in the FB condition than that in the TB condition, both in the overall analysis (McNemar Test, *p* = 0.008) and in the sequence FB-TB (McNemar Test, *p_total_* = 0.008, *p_sequence FB-TB_* = 0.031). These results, combined with the descriptive statistics, indicated that the verbal perceptual prompts promoted children to verbally differentiate between FB and TB, especially in the sequence FB-TB.

### 3.4. Proportion of Response Patterns

**Figure 6:**
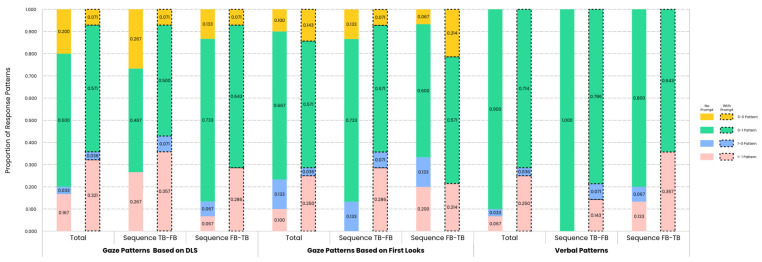
Proportion of gaze and verbal response patterns (FB-TB pattern) for the no-prompt group and the prompt group across trial sequences. In terms of gaze patterns based on DLS, children from the prompt group (indicated by dashed lines) exhibited an increased trend for the 1-1 gaze pattern in the sequence FB-TB. In terms of gaze patterns based on first looks, children from the prompt group exhibited significantly more 1-1 gaze patterns in the sequence TB-FB. In terms of verbal patterns, children from the prompt group also exhibited an increased trend for the 1-1 verbal pattern in the overall analysis and a decreased trend for the 0-1 verbal pattern both in the overall analysis and in the sequence TB-FB.

In pursuit of a broader spectrum of indicators from the perspective of differentiating between FB and TB scenarios to examine the effect of verbal perceptual prompts on children’s sensitivity to false beliefs, the results complemented the analysis of response patterns, which included gaze response patterns based on DLS, gaze response patterns based on first looks and verbal response patterns based on verbal answers. Descriptive results of the proportions of response patterns for the two groups are presented in Figure 6.

In terms of gaze patterns based on DLS, the direct comparison of proportions of the four gaze patterns between two groups revealed no significant group effect, and there was only anecdotal evidence for the alternative hypothesis or anecdotal to strong evidence for the null hypothesis (Fisher’s Exact Test, *p_s_* > 0.05; 0.697 ≤ BF_01_ ≤ 5.254), though descriptive statistics results showed an increased trend in the proportion of the 1-1 gaze pattern for the prompt group in the sequence FB-TB (from 6.7% to 28.6%), indicating a potential effect of verbal perceptual prompts to improve the likelihood of children exhibiting the commonly expected gaze position preference across the FB and TB conditions in the sequence FB-TB.

In terms of gaze patterns based on first looks, the direct comparison of proportions of the four gaze patterns revealed a significant group effect in the sequence TB-FB for the 1-1 gaze pattern. In the sequence TB-FB, the proportion of children from the prompt group who exhibited the 1-1 gaze pattern (28.6%) was significantly higher than that from the no-prompt group (0%), and there was moderate evidence for the alternative hypothesis (Fisher’s Exact Test, *p* = 0.042; BF_01_ = 0.232). The results suggested that the verbal perceptual prompts increased the likelihood for children to exhibit the commonly considered correct anticipatory looking behavior both under the FB condition and under the TB condition in the sequence TB-FB.

Similarly, in terms of verbal patterns, the direct comparison of proportions of the four verbal patterns revealed a marginally significant group effect in the overall analysis as well as in the sequence TB-FB. In the overall analysis, the proportion of children from the prompt group who exhibited the 0-1 verbal pattern (71.4%) was marginally significantly lower than that from the no-prompt group (90.0%), and there was anecdotal evidence for the alternative hypothesis (*χ*^2^ = 3.250, *df* = 1, *p* = 0.071; BF_01_ = 0.573). However, the proportion of children from the prompt group who exhibited the 1-1 verbal pattern (25.0%) was marginally significantly higher than that from the no-prompt group (6.7%), and there was anecdotal evidence for the alternative hypothesis (Fisher’s Exact Test, *p* = 0.054; BF_01_ = 0.500). Under the sequence TB-FB, the proportion of children from the prompt group who exhibited the 0-1 verbal pattern (*M* = 78.6%) was also marginally significantly lower than that from the no-prompt group (*M* = 100.0%), and there was anecdotal evidence for the alternative hypothesis (Fisher’s Exact Test, *p* = 0.100; BF_01_ = 0.549). These results, combined with the descriptive statistics, indicated that the verbal perceptual prompts reduced children’s tendency to consistently answer behavioral prediction questions based on objective reality across FB and TB conditions, especially in the sequence TB-FB, and helped children answer the behavioral prediction questions based on the protagonist’s beliefs across FB and TB conditions.

## 4. Discussion

In this study, a controlled experiment was designed to compare the performance of children on anticipatory looking tasks with and without verbal perceptual prompts. The findings revealed that children’s performance on anticipatory looking tasks was moderately better when provided with verbal perceptual prompts compared to tasks without such prompts. This suggests that verbal perceptual prompts can facilitate children’s sensitivity to false beliefs, although the effect remains relatively small.

### 4.1. The Role of Verbal Perceptual Prompts

Verbal perceptual prompts used in the present study served as reminders or response scaffolds to help the children make correct responses. The role of verbal perceptual prompts was manifested in the following ways. Regarding looking behaviors, verbal perceptual prompts reduced children’s preference for looking at the object’s actual location in the FB scenario, as indicated by an increase in DLS, and enhanced children’s tendency to exhibit an anticipatory looking pattern corresponding to the protagonist’s beliefs in both FB and TB scenarios, as indicated by the higher 1-1 looking pattern ratio. Regarding verbal responses, verbal perceptual prompts enhanced children’s tendency to correctly verbalize the empty position in the FB scenario, as indicated by higher verbal response accuracy, and enhanced their tendency to exhibit a verbal response pattern corresponding to the protagonist’s belief-based behavior expectation in both FB and TB scenarios, as indicated by the higher 1-1 verbal response pattern ratio. Additionally, they reduced children’s tendency to exhibit a verbal response pattern based on objective reality in both FB and TB scenarios, as indicated by the lower 0-1 verbal response pattern ratio. Overall, verbal perceptual prompts are beneficial for children’s attention to protagonists’ belief states and for their correct anticipation of the behaviors of protagonists with different belief states.

The findings of this study are consistent with previous research on children’s understanding of false beliefs, indicating that verbally related cues can serve as a scaffold to promote children’s attention to and their processing of others’ false beliefs (Hansen 2010; Siegal and Beattie 1991; Zhang and Zhou 2021). The results differ from those found by Kulke and Rakoczy, who did not find a consistent effect of verbal factors on children’s performance in anticipatory looking tasks (Kulke and Rakoczy 2019). This discrepancy may suggest that verbal factors promote children’s sensitivity to false beliefs only when the prompts genuinely address others’ belief content. In this study, the verbal content not only objectively narrated whether the protagonist was moved completely but also directly described the protagonist’s perceptual state as a result. This type of connection between an objective event and a perceptual state not only emphasizes the protagonist’s perceptual state but also provides a subtle demonstration of mental state reasoning. This approach guides children to continue considering the connection between the perceptual states and other mental states, such as beliefs.

This study also detected a sequence effect for the role of verbal perceptual prompts across trials, with the facilitation of children’s sensitivity to false beliefs particularly evident in the sequence TB-FB compared to the sequence FB-TB. This may be associated with the fact that the trial sequence also influenced how children’s sensitivity to false beliefs was expressed. In the absence of verbal perceptual prompts, children in the sequence TB-FB were more inclined to demonstrate gaze responses that contradicted the commonly considered correct anticipatory looking responses, responses that were based entirely on the anticipation of the protagonist’s actual behavior. Specifically, compared to the TB condition, in the FB condition, children from the no-prompt group showed a greater tendency to gaze at the full position with the object rather than the empty position without the object. This tendency may represent an anticipation of the protagonist’s need for assistance. That is to say, children may expect the protagonist with false beliefs to be more dependent on others’ help to achieve the goal of finding the object, leading them to gaze more at the object’s actual location, either to spontaneously help the protagonist fulfill the goal or correct the protagonist’s mistaken behavior. Such tendencies align with the adaptive helping behaviors shown by 18- and 24-month-old infants when facing protagonists with different belief states (Knudsen and Liszkowski 2012a, 2012b). When the task provided no prompts, initially presenting children with the TB scenario, which might have emphasized the achievement of goals, would have been more likely to trigger the form of children’s sensitivity to false beliefs associated with helping. The verbal perceptual prompts, when provided, were more readily interpreted by children as reminders oriented towards behavioral expectations. Consequently, in the sequence FB-TB, the verbal perceptual prompts were more likely to play a role in consolidating or strengthening the behavioral anticipatory form of children’s sensitivity to false beliefs. However, in the sequence TB-FB, the verbal perceptual prompts additionally contributed to shifting the manifestation of children’s sensitivity to false beliefs from a helping orientation to a behavioral anticipatory orientation, thereby making the facilitative effect of the verbal perceptual prompts on children’s sensitivity to false beliefs more evident in this sequence. 

### 4.2. The Manifestation of Children’s Sensitivity to False Beliefs

The results of this study differ from those of Kulke and Rakoczy (2019), which may also reflect issues in the choice of indicators for sensitivity to false beliefs. If the focus was solely on the accuracy of children’s anticipatory looking in the single FB scenario task, measured by the proportion of first looks at the empty position, this study similarly did not find a significant benefit of verbal factors on children’s sensitivity to false beliefs. The difference in the proportion of first looks at the empty position in the FB scenario task between the prompt group and the no-prompt group did not reach a significant level. However, when attention was paid to the difference in DLS and the difference in response patterns between FB and TB scenarios (especially the latter), the role of verbal factors became more apparent. Both the analysis of DLS and response patterns in different belief scenarios can be seen as indicators that emphasize differences, with the former focusing on the degree of response differences at different positions within a single belief scenario and the latter on the differences in response positions across different belief scenarios. Kulke and Rakoczy’s results, at least in terms of descriptive statistics, showed that the difference in the proportions of time children spent looking at different areas of interest in the anticipatory looking false belief task with verbal narration was larger than the corresponding difference in the task without verbal narration (Kulke and Rakoczy 2019). We posit that this larger difference also represents an enhancement of sensitivity to false beliefs.

It also needs to be noted that, even when focusing on difference-oriented indicators, we found that the sensitivity to false beliefs among children from the verbal perceptual prompt group was not outstanding. With additional verbal perceptual prompts, the proportion of children exhibiting the 1-1 correct anticipatory looking pattern was less than 30%. Moreover, if the 1-1 and 0-0 differential response patterns were combined, the total proportion of children exhibiting differential gaze patterns did not exceed 50%. This finding is somewhat similar to the mixed results regarding the replicability of sensitivity to false beliefs. It may suggest that there are other analytical methods or design approaches that are more effective in detecting children’s sensitivity to false beliefs. Recently, some studies have used multi-trial anticipatory looking false belief tasks, employing indicators that combine performance across multiple FB trials, and have obtained completely positive (Grosse Wiesmann et al. 2017) or partially positive (Kaltefleiter et al. 2022) verification results. Combining these positive verification results, our findings support the view that children’s sensitivity to false beliefs is relatively difficult to detect (Baillargeon et al. 2018).

### 4.3. Limitation and Future Directions

The present study revealed that verbal perceptual prompts can facilitate children’s sensitivity to false beliefs, yet the results also indicate that the effect of such prompts is relatively small. This may suggest that verbal perceptual prompts are not the decisive factor in triggering a robust sensitivity to false beliefs in children, and future research could explore other factors with a greater effect. On the other hand, the results may also reflect the limitations of the experimental design in this study. The task employed more variable protagonists and target objects, which, while effectively capturing children’s attention and increasing their motivation to participate in the experiment, might have somewhat reduced their focus on the protagonist’s false beliefs, with the verbal perceptual prompts only having a limited compensatory effect. Future studies could design tasks that more effectively stimulate both the participants’ motivation to engage in the experiment and their motivation to focus on others’ false-belief states in order to reexamine the role of verbal perceptual prompts. A meta-analysis of 33 articles on infants’ sensitivity to false beliefs showed that infants performed better in the violation-of-expectation paradigm than in the anticipatory looking paradigm, and as infants got older, the reported pass rates in studies increased (Barone et al. 2019), indicating differences in paradigms and age. Therefore, future research could also examine the role of verbal perceptual prompts in sensitivity to false beliefs with other paradigms and in other developmental stages.

### 4.4. Implications

Beyond the previously discussed issues of indicator selection, this study suggests that the development of children’s sensitivity to false beliefs may be situation-dependent. The inclusion of a simple verbal statement regarding the protagonist’s perceptual state within the task situation can significantly influence children’s performance in tasks assessing sensitivity to false beliefs. This aligns with the view of the situational mental file account, which posits that the development of sensitivity to false beliefs is closely related to situational factors, and factors which focus on the protagonist instead of the object can promote the manifestation of sensitivity to false beliefs (Newen and Wolf 2020). However, theoretically, the verbal perceptual prompts in this study provide a reminder to focus on the protagonist’s mental state, yet their enhancing effect on children’s sensitivity to false beliefs is relatively small. This suggests that in the anticipatory looking task of this study, the lack of a significant demonstration of children’s sensitivity to false beliefs is not due to a lack of understanding the perceptual cues in the task situation or difficulty in inferring the protagonist’s perceptual state. What situational factors are more critical for the manifestation of children’s sensitivity to false beliefs still requires careful examination. In terms of the situation-dependence of children’s sensitivity to false beliefs, this study suggests that research on sensitivity to false beliefs can adopt a cue-based approach to the ToM, focusing on examining what range of stimulus conditions and task contexts give rise to different characteristics of the ToM systems (German and Cohen 2012). Even though sensitivity to false beliefs is more often manifested as an implicit and spontaneous process, it still requires appropriate cues for triggering. The findings also offer insights for educational practice, suggesting that to enhance children’s spontaneous attention to others’ mental states, social contexts enriched with cues triggering such attention can be created. Just as scaffolds in general intelligence education support the development of children’s general intelligence, appropriate scaffolds in social intelligence education can facilitate the development of children’s social intelligence.

To sum up, our results demonstrated a dissociation between children’s performance on anticipatory looking tasks with and without verbal perceptual prompts, which indicates that verbal perceptual prompts can facilitate the manifestation of children’s sensitivity to false beliefs. The results highlight the situation-dependent characteristics of children’s sensitivity to false beliefs. Future research could investigate the situational factors that may more effectively trigger a robust sensitivity to false beliefs in children across different paradigms and age groups.

## Data Availability

The data presented in this study will be made available by the authors on request due to privacy and ethical reasons.
